# Effects of Photodynamic Therapy on Tumor Metabolism and Oxygenation Revealed by Fluorescence and Phosphorescence Lifetime Imaging

**DOI:** 10.3390/ijms25031703

**Published:** 2024-01-30

**Authors:** Marina V. Shirmanova, Maria M. Lukina, Marina A. Sirotkina, Liubov E. Shimolina, Varvara V. Dudenkova, Nadezhda I. Ignatova, Seiji Tobita, Vladislav I. Shcheslavskiy, Elena V. Zagaynova

**Affiliations:** 1 Institute of Experimental Oncology and Biomedical Technologies, Privolzhsky Research Medical University, Minin and Pozharsky Sq. 10/1, 603005 Nizhny Novgorod, Russia; 2 Lopukhin Federal Research and Clinical Center of Physical-Chemical Medicine of Federal Medical Biological Agency, Malaya Pirogovskaya, 1a, 119435 Moscow, Russia; 3 Department of Chemistry and Chemical Biology, Gunma University, Kiryu 376-8515, Gunma, Japan

**Keywords:** photodynamic therapy, fluorescence lifetime imaging FLIM, phosphorescence lifetime imaging PLIM, metabolism, oxygenation, KillerRed, Photoditazine

## Abstract

This work was aimed at the complex analysis of the metabolic and oxygen statuses of tumors in vivo after photodynamic therapy (PDT). Studies were conducted on mouse tumor model using two types of photosensitizers—chlorin e6-based drug Photoditazine predominantly targeted to the vasculature and genetically encoded photosensitizer KillerRed targeted to the chromatin. Metabolism of tumor cells was assessed by the fluorescence lifetime of the metabolic redox-cofactor NAD(P)H, using fluorescence lifetime imaging. Oxygen content was assessed using phosphorescence lifetime macro-imaging with an oxygen-sensitive probe. For visualization of the perfused microvasculature, an optical coherence tomography-based angiography was used. It was found that PDT induces different alterations in cellular metabolism, depending on the degree of oxygen depletion. Moderate decrease in oxygen in the case of KillerRed was accompanied by an increase in the fraction of free NAD(P)H, an indicator of glycolytic switch, early after the treatment. Severe hypoxia after PDT with Photoditazine resulted from a vascular shutdown yielded in a persistent increase in protein-bound (mitochondrial) fraction of NAD(P)H. These findings improve our understanding of physiological mechanisms of PDT in cellular and vascular modes and can be useful to develop new approaches to monitoring its efficacy.

## 1. Introduction

Photodynamic therapy (PDT) is a tumor treatment modality based on the ability photosensitive substances—photosensitizers—under local exposure to laser irradiation generate reactive oxygen species that cause the death of tumor cells [1,2]. The anti-tumor effect of PDT is based on three mechanisms: (1) direct phototoxic damage to tumor cells; (2) vascular damage; and (3) activation of a non-specific immune response [3]. The relative contribution of each of them depends on many factors: the chemistry of the photosensitizer, its localization in the tumor, the degree of vascularization and the content of macrophages in the tumor, the time from the injection of the photosensitizer to irradiation, etc. The predominance of the cellular mechanism should be expected with a high concentration of the photosensitizer in tumor cells and low concentration in the blood, which is typically achieved at a long drug-light interval. Vascular mechanism of PDT prevails when photodynamic reactions caused by the sensitizer target the tumor vessels, which lead to vascular stasis, thrombosis, hemorrhage, hypoxia and, as a consequence, death of tumor cells [4,5]. This is observed either with specific vascular-targeted photosensitizers or at short drug-light intervals with common photosensitizers. In practice, many PDT regimens suggest the realization of both cellular and vascular effects concurrently.

Although PDT is firmly established in clinical practice, some of its biological aspects remain poorly investigated, specifically its effects on tumor metabolism. Glycolytic phenotype is considered as a factor of poor prognosis for patients undergoing PDT, and pharmacological inhibition of glycolysis increases its effectiveness [5]. At the same time, the hypoxia and oxidative stress induced by PDT in the tumor can promote the metabolic switch to glycolysis and the activation of molecular pathways (primarily the hypoxia-inducible factor-1, HIF-1) leading to the survival of more aggressive tumor cells [6,7]. It is assumed that the metabolic response of the tumor depends on the mechanisms of action of PDT and differs for the drugs causing a direct cell kill or vascular shutdown. For example, the differences in tumor glucose uptake profiles between the two PDT protocols have been identified by positron emission tomography with ^18^F-fluorodeoxyglucose; a rapid decrease in glucose uptake followed by a rapid recovery was observed in the case of cellular mode and a delayed decrease in glucose levels and recovery to significantly lower levels—in the case of vascular mode [8]. In cellular-targeted PDT metabolic alterations are often associated with mitochondrial damage, which results in the reduction in adenosine triphosphate level and triggers mitochondrial production of reactive oxygen species (ROS), which in turn leads to apoptotic cell death [9,10]. Metabolomic data suggest that PDT affects various components of glycolysis and the citric acid cycle as well as metabolites involved in redox signaling. The metabolic processes that are dependent on mitochondria were downregulated, whereas the antioxidant response was activated after PDT with liposomal zinc phthalocyanine in vitro [11]. Metabolic transitions after vascular-targeted PDT are, most likely, due to blood flow stasis and hypoxia as well as nutrient deprivation. In any PDT mode, oxidative stress is induced by generation of free radicals, which is closely linked to cellular metabolic profile [12]. However, the associations of metabolic reactions with the changes in tumor oxygenation and redox state are not well characterized. In vivo studies with parallel monitoring of tumor metabolism and oxygen in the course of PDT are especially lacking.

Modern optical techniques such as combined fluorescence and phosphorescence lifetime imaging (FLIM and PLIM, correspondingly) provide a unique opportunity to monitor cellular metabolism and tissue oxygenation non-invasively, in tumor models in vivo. Probing of metabolism using FLIM relies on the recording of endogenous fluorescence of the redox-cofactors nicotinamide adenine dinucleotide (phosphate) NAD(P)H in the reduced state and flavin adenine dinucleotide FAD in the oxidized state that act as electron donor and acceptor in reactions of energy metabolism [13]. The free form of NAD(P)H associated with glycolysis has short fluorescence lifetime (~0.4 ns), while the protein-bound form, associated with mitochondrial oxidative phosphorylation has longer lifetimes (1.7–3.5 ns) [14]. Thus, by extracting the relative contribution of the short and long components from the decay curve upon bi-exponential fitting, it is possible to conclude about the changes in the balance between glycolysis and oxidative metabolism. Unlike NAD(P)H, FAD fluorescence decay has a more difficult interpretation and its fluorescence intensity is typically low in the tumors, so it is rarely used as a metabolic indicator. Given a label-free principle of contrast acquisition, a high sensitivity and molecular specificity of NAD(P)H FLIM, it is considered as a valuable research tool with a great potential for clinical use [15].

PLIM allows for assessing the molecular oxygen content in a tumor using oxygen-sensitive phosphorescent probes. Bimolecular collisions of the probe with molecular oxygen shorten the probe’s triplet lifetime and quench phosphorescence [16,17], so that the phosphorescence decay time of the probe linearly decreases with the increase in oxygen concentration, according to the Stern–Volmer equation. The typical phosphorescent probes are the synthetic organic complexes with transition metals, such as Pt(II), Pd(II), Ru(II), and Ir(III). While numerous oxygen probes have been developed so far, only few of them are suitable for in vivo applications. Depending on the location of the probe within tumor tissue, oxygen concentration can be assessed inside the blood vessels, in the interstitial space and/or inside the cells [18,19,20]. Due to much longer phosphorescence decay time (µs to ms) compared to fluorescence, the measurements of oxygen can be combined with fluorescence imaging, including NAD(P)H FLIM [21,22]. 

The purpose of this work was to investigate the relationships between the changes in oxygenation and metabolic activity of tumors in vivo induced by PDT in “cellular” and “vascular” modes of action. For “cellular” PDT, the genetically encoded photosensitizer KillerRed was used, and vascular-targeted PDT was carried out with chlorin e6 derivative Photoditazine. Here, we implemented fluorescence lifetime imaging of NAD(P)H on a two-photon laser scanning microscope and phosphorescence lifetime imaging with the oxygen probe BTPDM1 on a one-photon confocal macroscanner [23]. Additionally, intravital imaging of tumor microvasculature was performed after vascular PDT using optical coherence tomography-based angiography (OCA). Therapeutic efficacy of both PDT protocols was confirmed by the inhibition of tumor growth and histopathological alterations.

## 2. Results

### 2.1. Metabolic Changes after PDT

Using NAD(P)H FLIM, data were obtained on changes in the metabolic status of tumor cells after PDT with genetically encoded photosensitizer KillerRed or Photoditazine. 

**Figure 1:**
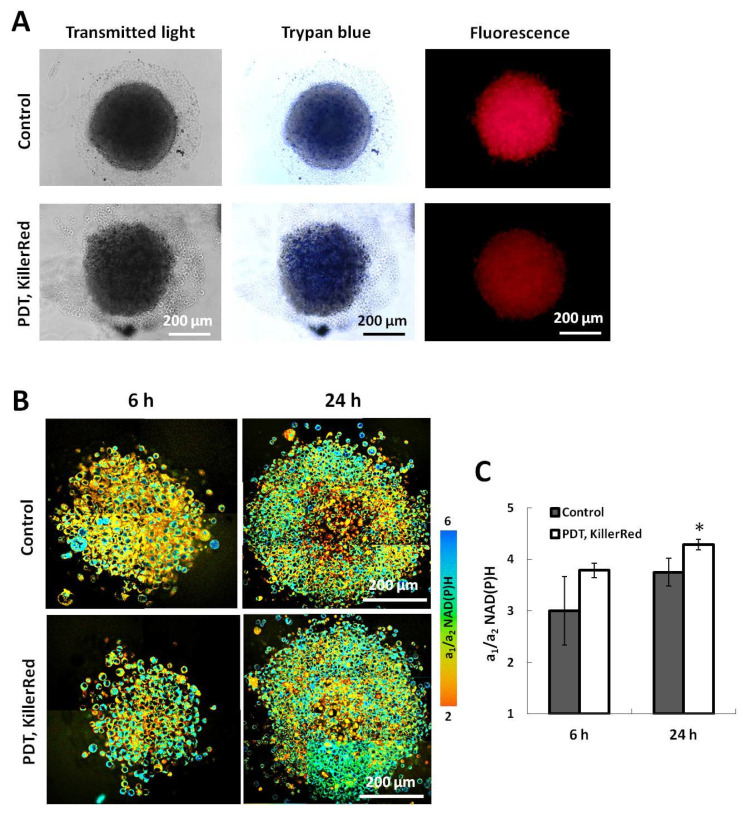
Effects of PDT with KillerRed on multicellular tumor spheroids. (**A**) Changes in spheroid structure and cell viability in 24 h and in fluorescence intensity immediately after PDT. (**B**) FLIM-microscopy of NAD(P)H in control and PDT-treated spheroids at 6 h and 24 h time points. (**C**) Quantification of NAD(P)H a_1_/a_2_ value in spheroid’s cells. *—statistically significant differences with control at the same time point (*p* ≤ 0.05). Mean ± SD, *n* = 4–5 spheroids, 30–40 cells in each. NAD(P)H FLIM measurements were performed only in viable cells within spheroids. Scale bar 200 µm.

At the first step of the study of metabolic effects of “cellular” PDT with KillerRed, the experiments on the tumor spheroids were performed (Figure 1). Control spheroids were 400–500 µm in diameter and had a typical dense structure. They consisted of the thin outer layer of proliferating cells, middle layer of quiescent cells and necrotic core. PDT (50 mW/cm^2^, 25 min, 75 J/cm^2^) caused alteration of spheroids morphology—the proportion of dead (trypan blue stained) cells increased, and they were distributed across the whole spheroid; the spheroids became loosely packed and weakly adhered to the dish bottom. The photodynamic effects of KillerRed were accompanied by its photobleaching by ~50% at the regimen used, which is consistent with our previous results [24]. FLIM of NAD(P)H revealed the increased a_1_/a_2_ ratio in PDT-treated compared with untreated spheroids at 6–24 h post-PDT (4.28 ± 0.10 vs. 3.75 ± 0.27, *p* = 0.005) suggesting the glycolytic shift in cellular metabolism (Figure 1).

**Figure 2:**
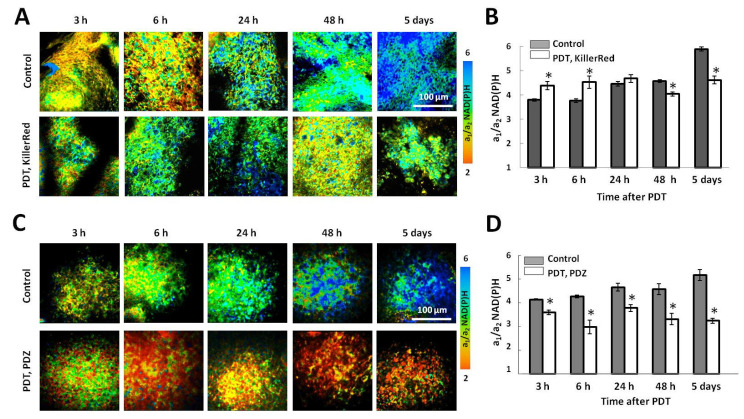
In vivo study of the metabolic status of mouse tumors after PDT using NAD(P)H FLIM. Representative microscopic FLIM images of control tumors and tumors after PDT with the genetically encoded photosensitizer KillerRed located in the cell nuclei (**A**) or Photoditazine (**C**). The ratios of the free to protein-bound forms of NAD(P)H a_1_/a_2_ are shown. Time after treatment is indicated above the images. Scale bar 100 µm. Quantification of NAD(P)H a_1_/a_2_ value (**B**,**D**). *—statistically significant differences with control at the same time point (*p* ≤ 0.05). Mean ± SEM, *n* = 4–10 tumors.

In mouse tumors, PDT with KillerRed resulted in the increased NAD(P)H a_1_/a_2_ ratio at early time points (3–6 h) compare to the control (4.38 ± 0.17 vs. 3.79 ± 0.05, *p* = 0.011), indicating that the treated tumors were more glycolytic (Figure 2A,B). At later times, 2–5 days, the NAD(P)H a_1_/a_2_ ratio in the treated group was statistically lower than in control. These results obtained on mouse tumors in vivo are consistent with the data obtained on tumor spheroids.

In the control groups, the a_1_/a_2_ ratio of NAD(P)H gradually increased during 5 days of tumor growth from ~4 to ~5.5, which indicated a metabolic shift towards glycolysis. 

In the group of “vascular” PDT with Photoditazine all tumors had statistically reduced NAD(P)H a_1_/a_2_ ratio compared to untreated controls (3.52 ± 0.056 vs. 4.14 ± 0.018, *p* = 0.013) already 3 h after laser irradiation. Upon further observation during 5 days, the differences between control and treated tumors’ metabolism became more pronounced, mainly due to increase in the free NAD(P)H (a_1_) pool in the control tumors (Figure 2C,D).

Analysis of the fluorescence lifetime of NAD(P)H in tumor cells after PDT with either of the photosensitizers did not reveal statistically significant changes. The value of the short component (a_1_) corresponding to free NAD(P)H was ~0.4 ns and of the long component (a_2_) corresponding to protein-bound NAD(P)H was ~2.5 ns both in spheroids and tumors in vivo (Table S1).

Therefore, our NAD(P)H FLIM study showed that PDT can cause different metabolic responses in the tumors in vivo, including both the elevation of the contribution from glycolytic, free, NAD(P)H pools and the increase in mitochondrial, bound NAD(P)H fraction.

### 2.2. Tumor Oxygenation after PDT

In order to assess the oxygenation status of the tumors after PDT, the macroscopic PLIM with phosphorescent oxygen probe BTPDM1 was performed on the same tumors as NAD(P)H FLIM.

Untreated KillerRed-expressing tumors had phosphorescence lifetime of BTPDM1 ~3.86 μs. In naïve tumors (control for PDT with Photoditazine), the initial phosphorescence lifetime was ~4.96 μs indicating their slightly worse oxygenation compared with tumors expressing KillerRed. The differences in oxygen status between the control groups are likely due to the fact that they were examined at different time points after tumor inoculation (Day 13 for KillerRed-expressing tumors, Day 7 for naïve tumors).

**Figure 3:**
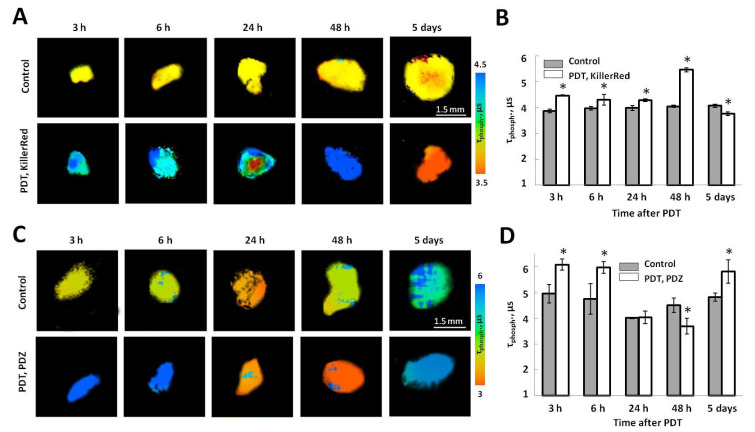
In vivo assessment of the oxygen status of mouse tumors after PDT using PLIM. Representative macro-PLIM images of control tumors and tumors after PDT with the genetically encoded photosensitizer KillerRed located in the cell nuclei (**A**) or Photoditazine (**C**). Time after treatment is indicated above the images. Scale bar 1.5 mm. Note, that images in A and C are shown in different scales. (**C**) Phosphorescence lifetimes of the BPTDM1 oxygen-sensitive probe after PDT (**B**,**D**). *—statistically significant differences with control at the same time point (*p* ≤ 0.05). Mean ± SEM, *n* = 3–4 tumors.

In the case of PDT with KillerRed, the BTPDM1 phosphorescence lifetimes were statistically higher in the period from 3 h to 48 h, but during first 24 h these changes were less pronounced than after PDT with Photoditazine and did not exceed 0.5 μs. The greatest difference in the oxygen status of treated and untreated tumors was recorded 48 h after PDT (5.46 ± 0.07 vs. 4.05 ± 0.05 μs, *p* = 0.003). In 5 days after PDT, BPTDM1 phosphorescence lifetime was shorter compared to control tumors (3.77 ± 0.07 vs. 4.19 ± 0.06 μs, *p* = 0.011), which indicated reoxygenation of the treated tumors (Figure 3A,B).

It was found that in the time-period from 3 to 6 h after PDT with Photoditazine, the phosphorescence lifetime of BTPDM1 in the tumors was significantly longer than in untreated control (6.09 ± 0.21 μs vs. 4.96 ± 0.35 μs at 3 h, *p* = 0.001), which indicate the development of hypoxia in the tumor. Then, in 24 h and 48 h after irradiation, a decrease in the lifetime of BTPDM1 was recorded, which may be associated with reoxygenation of the tumor tissue. However, 5 days after PDT, the oxygen level in the treated tumors was again lower than in control (Figure 3C,D).

Since the genetically encoded photosensitizer KillerRed is expressed by the tumor cells themselves and does not re-distribute within the tumor tissue, the reduced oxygenation detected by PLIM after PDT can be attributed exclusively to the consumption of oxygen for photodynamic reactions, at least at the early time points. PDT with Photoditazine in the regimen used causes vascular damages; therefore, it can be assumed that changes in the oxygen content could be due to both, the oxygen consumption and stopping the supply of oxygen to the tumor cells.

### 2.3. Antivascular Effects of PDT with Photoditazine

**Figure 4:**
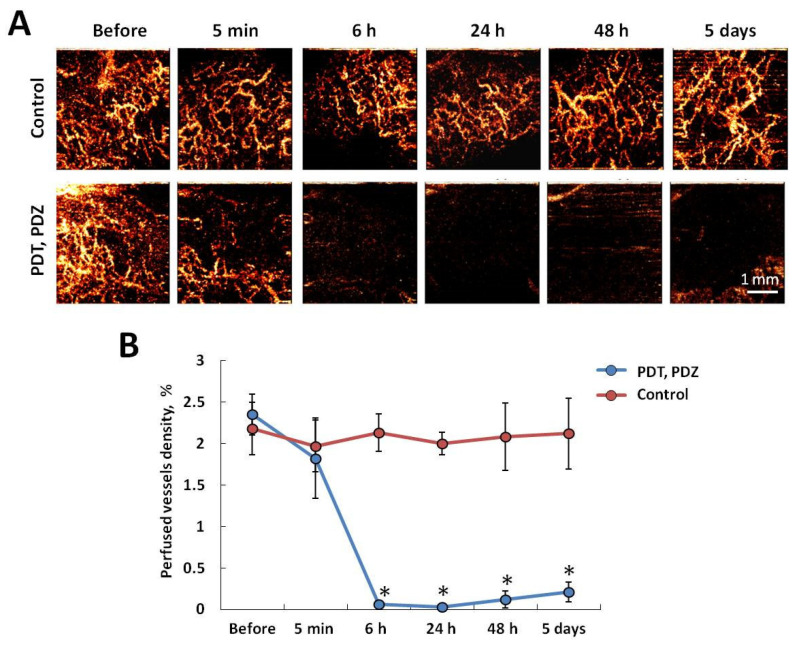
In vivo imaging of the perfused blood vessels in mouse tumors after PDT with Photoditazine using OCT-based angiography. (**A**) Representative OCA images of vascular network in the control or treated tumors on the indicated time points after PDT. A maximum intensity projection 2D display represents 3D data to a depth of 1.3 mm. Bar is 1 mm, applicable for all images. (**B**) Quantification of the perfused vessels density in the control and treated tumors. Mean ± SEM, *n* = 5–8 tumors. *—statistically significant differences with control at the same time point (*p* ≤ 0.05).

Dynamic observation of the tumor vascular response to PDT with Photoditazine was carried out using the OCA in vivo in parallel with oxygen mapping by PLIM (Figure 4). Before PDT, all tumors were characterized by a dense vascular network consisting of thin, tortuosity vessels. In the control group, throughout the entire observation period, the structure of the vascular network and its density did not change. Immediately after PDT, only local vessel reactions were observed in some of the tumors. In 6 h after PDT, in three out of eight animals, there were no visible vessels on OCA images; in the remaining five tumors, the density of the vascular network significantly decreased to values close to 0. After 24 h and 48 h, blood vessels were not visualized on OCA images in all tumors. By the 5th day after PDT, perfused vessels appeared at the edges of some tumors, probably due to their re-growth from the peri-tumorous tissue.

Therefore, monitoring of the vascular response of the CT26 tumors to PDT with Photoditazine revealed a complete stop of blood flow in 6–48 h after PDT, which was manifested as the absence of visible blood vessels on the OCA images (*p* < 10^−5^ with control) and suggested an irreversible strong vessel reaction.

Since the measurements of oxygen and microvasculature were performed from the same individual tumors, we have made an attempt to correlate these variables with each other. Plotting oxygen content (BTPDM1 phosphorescence lifetime) against perfused vessels densities showed no associations for both untreated (r = 0.2892) and PDT-treated tumors (r = 0.1204) (Figure S1). Both well- and poorly-vascularized tumors could be oxygenated equally. This suggests that different factors, besides vessel density, determine oxygen concentration in the tissue, at least within the tumor growth stage included in the study.

### 2.4. Verification of Anti-Tumor Effects of PDT

**Figure 5:**
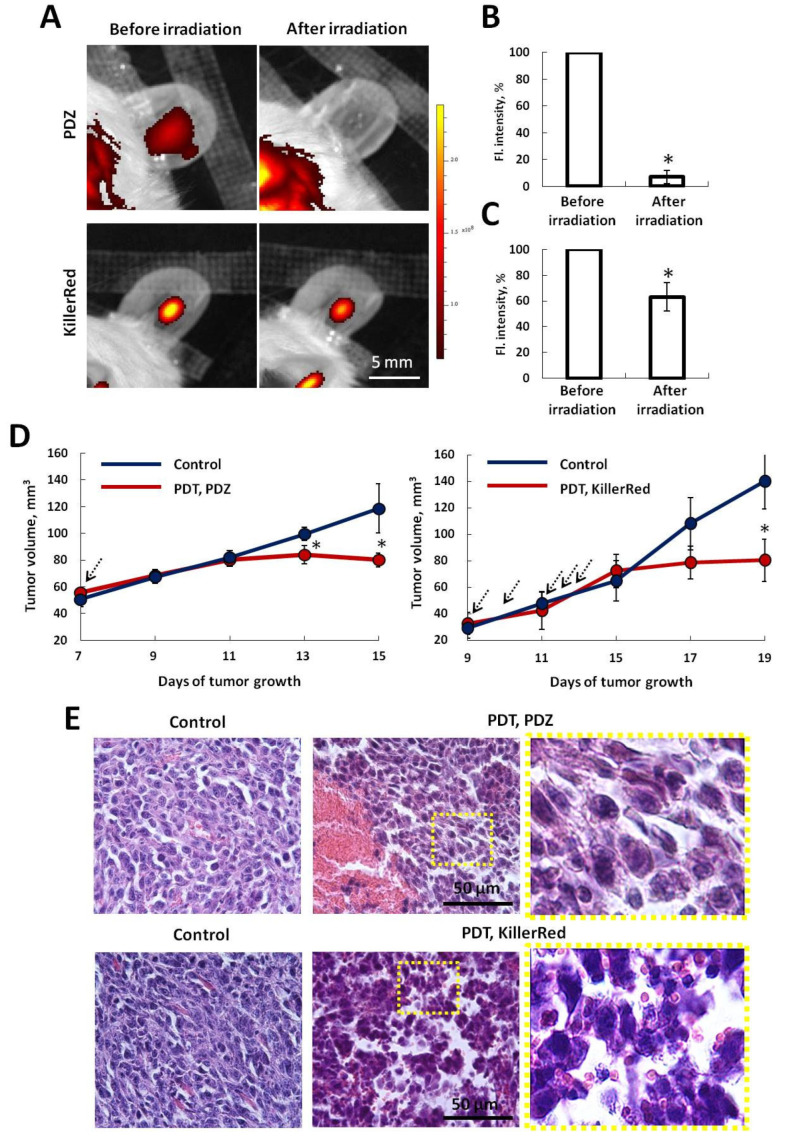
The effects of PDT on the fluorescence, growth rate and histopathological structure of the CT26 and CT26-KillerRed tumors. (**A**) Photobleaching of the photosensitizers after PDT. In vivo fluorescence intensity images of tumors are shown. Scale bar 5 mm. Quantification of the fluorescence intensity in the tumors after PDT with Photoditazine (PDZ) (**B**) or KillerRed (**C**). Mean ± SD, *n* = 4–5 tumors. For KillerRed fluorescence intensity before and after the first irradiation procedure is shown. *—statistically significant differences with control before irradiation (*p* ≤ 0.05). (**D**) Monitoring of tumor volume in control and treated groups. Mean ± SEM, *n* = 4–10 tumors. *—statistically significant differences with control at the same time point (*p* ≤ 0.05). PDT procedures are indicated by the arrows. (**E**) Histopathology of the control and treated tumors on the 5th day after PDT. H&E. Initial magnification ×40. Scale bar: 50 µm. Enlarged areas (right) are shown in the dashed squares.

Fluorescence intensity imaging of tumors in vivo was performed before and after laser irradiation to assess photobleaching of photosensitizers, an indirect indicator of treatment efficacy (Figure 5A). The irradiation regimens used for PDT caused ~90% decrease in the fluorescence intensity in the case of Photoditazine and ~40% in the case of KillerRed (Figure 5B,C). These fluorescence measurements showed that PDT with Photoditazine is likely more efficient in terms of ROS generation, which allowed us to optimize PDT dosimetry, specifically the number of irradiation procedures.

The therapeutic effects of PDT with Photoditazine or KillerRed on the CT26 mouse tumors were confirmed by inhibition of tumor growth and pathomorphological disorders (Figure 5D,E). However, in order to achieve these effects with KillerRed multiple (x5) irradiation of tumors was required with a rather high light dose compared with Photoditazine.

It was shown that after PDT with Photoditazine, tumors inhibited their growth starting from the 13th day of growth (6 days after PDT, *p* = 0.01 with control). Once the treated tumors reached a volume of 75–80 mm^3^ on the 11th day of growth, their sizes did not change throughout the entire observation period until the 19th day. Whereas the untreated tumors grew actively, and their size increased from ~50 mm^3^ on the 7th day to ~110 mm^3^ on the 15th day after inoculation (Figure 5D).

Analysis of tumor growth after PDT with the genetically encoded photosensitizer KillerRed showed that it led to inhibition of tumor growth starting from the 17th day (4 days after PDT). On the 19th day, the differences between the treated and untreated tumor sizes were statistically significant (*p* = 0.002) (Figure 5D).

Histological analysis showed that control CT26 tumors, both naïve and expressing KillerRed, had typical structure with high mitotic activity, and the content of viable cells was 90–100% (Figure 5E). The cells had round or oval large nuclei, predominantly with a diffuse distribution of chromatin and 1–2 nucleoli. Dystrophic changes in cells and apoptosis were rare. The areas of spontaneous necrosis did not exceed 5%.

PDT with Photoditazine yielded in massive necrosis of tumor tissue (up to 70–80% of the tumor area) and pronounced vascular reaction with hemorrhages and hemolysis, which was observed in 5 days after PDT. The cellular component of the tumor was sparse, the boundaries of tumor cells were blurred and difficult to identify. Tumor cells in the viable part of the tissue were characterized by pronounced polymorphism, nuclear edema, the loss of integrity of the cell membranes. Similarly, after PDT with KillerRed the total destruction of the tumor tissue and massive necrosis were revealed. Single viable cells had serious dystrophic changes in the form of disruption of membrane integrity, chromatin condensation, cellular edema, and blurring of cell boundaries (Figure 5E).

Therefore, both PDT regimens were effective in the CT26 tumors in mice.

## 3. Discussion

Metabolic reorganizations in tumors as an effect of PDT have attracted increasing attention in the past decade, but our knowledge of this aspect of PDT remains very limited. Here, we attempted to identify the relationships between energy metabolism, the level of oxygenation and the result of PDT in vivo using the following optical imaging approaches: (1) two-photon fluorescence lifetime microscopy of NAD(P)H to monitor the cellular metabolic status of cells within tumors, (2) macroscopic PLIM with oxygen-sensitive probe to monitor the oxygen status of the tumors, and (3) OCT-angiography to verify the effects of PDT on the blood perfusion in the case of vascular-targeted mode. A comprehensive in vivo study on mouse tumor model CT26 was performed for two PDT modalities—cellular, using the genetically encoded photosensitizer KillerRed, and vascular, using chlorin e6–based photosensitizer Photoditazine.

Since oxygen is directly involved in photochemical reactions in PDT, an evaluation of the initial oxygen status of the tumor is essential for the effective implementation of the treatment [25]. On the other hand, PDT leads to a depletion of oxygen in the tumor, which may have unfavorable consequences, such as activation of angiogenic pathways and surveillance of the most aggressive populations of tumor cells that had been adapted to hypoxic environment [7]. Therefore, evaluating oxygen distribution in the tumor may help in the optimization of treatment protocols. It is known that hypoxia following PDT can arise either from the consumption of molecular oxygen directly for photochemical reaction with photosensitizer or from damage to the microvasculature resulting in a significant decrease in the blood flow, or both, depending on the PDT regimen [26]. As anticipated, both the cellular and vascular PDT modalities caused a decrease in the oxygen content in tumor cells early (within 3 h) after therapy; however, in the case of vascular PDT with Photoditazine hypoxia was more pronounced (Figure 3). The development of hypoxia as a result of PDT with KillerRed is associated exclusively with the consumption of oxygen for photodynamic reactions, while hypoxia after PDT with Photoditazine could result from both consumption of oxygen and vascular shutdown. Further changes in oxygen status differed: in the case of cellular PDT, the development of hypoxia was observed (possibly due to an increase in oxygen consumption by cells) followed by reoxygenation; in the case of vascular PDT, reoxygenation preceded secondary hypoxia resulting from irreversible vascular damage (Figure 3).

To monitor oxygen status of tumors we used PLIM with phosphorescent oxygen probe BTPDM1. According to Yoshihara et al. BTPDM1 has a high cellular uptake efficiency in cultured cells and re-localizes from the blood to the tumors tissues within a short period after intravenous injection [27]. A good cell- and tissue-penetrating ability of BTPDM1 allowed us to assess tissue oxygenation with this probe upon its local injection directly to the tumor. Earlier, PLIM was used for oxygen measurements in only a few works related to PDT. Kalinina et al. presented the PLIM-FLIM study of oxygen consumption and the cellular metabolic state during PDT with the TLD1433 agent that is simultaneously a photosensitizer and a phosphorescent oxygen probe. Using two-photon PLIM-FLIM-microscopy, they showed on the human urinary bladder carcinoma cells T24 in vitro elongation of phosphorescence lifetimes after PDT, an indication of low oxygen concentration, and a shortening of the fluorescence lifetime of NAD(P)H, an indication for glycolytic shift [28]. Their result corroborates our observation of lower oxygen and greater free NAD(P)H fraction after PDT with KillerRed. In the study by Stepinac et al. a porphyrin dye PdTCPP was simultaneously used as an oxygen sensor and a photosensitizer in vivo on the optic disc of piglets [29]. Photoirradiation induced alterations of the vascular endothelium and the increase in phosphorescence lifetime (i.e., the depletion of O_2_).

Vascular effects of PDT with chlorin e6-based photosensitizers are well documented. For example, Dong et al. performed hemodynamic monitoring of chlorin e6-mediated PDT in mice with mammary tumor EMT-6 using diffuse optical spectroscopy; the authors observed a decrease in relative blood flow and tissue oxygenation in responders starting from 3 h post-PDT without recovery up until 48 h [30]. Saito et al. analyzed vascular changes after PDT with Mono-L-aspartyl Chlorin e6 in fibrosarcoma-bearing mice and found relatively marked vascular degeneration and blood stasis in 4 h after irradiation at 10 min and 2 h drug-light intervals [31]. The in vivo study by Kirillin et al. using the optical coherence angiography on mice with CT26 tumors demonstrated vasculature response to PDT after intravenous injection of a chlorin e6-based photosensitizer Photolon but not with topical application of Revixan [32]. In accordance with our PLIM results, a rapid decrease in blood oxygen saturation was revealed using diffuse optical spectroscopy in CT26 mouse tumors upon PDT with Photolon, which was explained by the blood flow arrest [33]. Our previous studies using Photoditazine showed early (within 24 h post PDT) microvascular damage in CT26 tumors, which was detected by the optical coherence angiography [34]. Notably, in non-responders the blood flow partially recovered in 24 h post PDT, unlike responders. In a model of chorioallantoic membrane, Buzza et al. observed more pronounced vascular effects of PDT with Photodithazine compared with porphyrin-based compound Photogem [35]. At the same time, Photoditazine is uptaken by cancer cells in vitro [36] and in vivo (at longer accumulation times) [37], so direct cytotoxic effects can be also induced. However, “cellular” mode of PDT with Photoditazine was out of the scope of this study and will be examined further.

Previous studies of others and our groups provided evidence that KillerRed is capable to induce oxidative damage to the tumor cells in different models—monolayer cultures [38], multicellular spheroids [23] and tumor xenografts [39]. However, compared to traditional chemical photosensitizers, it has lower phototoxicity and thus requires higher light doses and multiple irradiations, especially in vivo, which along with the need for gene delivery, makes the prospects for clinical use of KillerRed, at least as a monotherapy, in the nearest future rather vague. But taking into account the unique advantages of KillerRed, it is considered as a promising tool for research of cellular responses to PDT [40]. Its red-shifted fluorescence emission makes it possible to perform a combined imaging with endogenous fluorescence of metabolic cofactors NAD(P)H and flavins and, therefore, to gain an insight into metabolic mechanisms of cellular-targeted PDT. This opportunity was first demonstrated by Lin et al. [41]. The authors assessed autofluorescence intensity of NAD(P)H and flavins in tumors’ cryo-sections and showed that NAD(P)H and flavoproteins were oxidized in the course of KillerRed-based PDT. Our study is, therefore, the first that exploited NAD(P)H fluorescence in time-resolved mode to monitor metabolic changes in response to PDT with KillerRed in vivo.

In our study, different metabolic responses were observed after PDT with Photoditazine (Type II, vascular mode) and KillerRed (Type I, cellular mode). In the case of Photoditazine, the ratio of free/bound NAD(P)H forms was stably lower than in control, which usually testifies to a shift to an oxidative metabolism. In line with our findings, Broekgaarden et al. have observed an increased FAD/(NADH + FAD) optical redox ratio in 3D culture model of pancreatic cancer after PDT with a benzoporphyrin derivative, which the authors attributed to severe oxidative stress [42]. In contrast, PDT with KillerRed resulted in increased free/bound NAD(P)H ratio early (1–6 h) after PDT, indicating a more glycolytic tumor state. Later on, the ratio did not change, but was statistically lower than in control tumors, that became more glycolytic during natural growth.

Given a development of hypoxia in the tumor tissue after both PDT regimens, as followed from concurrent PLIM measurements, a shift to an oxidative metabolism after vascular PDT with Photoditazine was quite an unexpected result. However, we noticed that oxygen depletion after this regimen was more marked than after PDT with KillerRed, which can explain the differences in the metabolic responses. Decreasing oxygen tension elicits different alterations in cellular metabolism and redox state depending on severity and duration of hypoxia [43]. Upon acute or mild hypoxia, metabolic adaptation takes a place—HIF-1α accumulates in the cytoplasm, translocates to the nucleus and promotes the expression of various metabolism-related genes and activation of vascular endothelial growth factor (VEGF), thus accelerating anaerobic glycolysis and angiogenesis [44,45]. Chronic or severe hypoxia alleviates ROS generation in the mitochondrial electron transport chain that causes oxidative stress [46]. At that, reducing equivalents (mostly NADH and FADH_2_) in the mitochondria are elevated owing to slowing of electron transport and consequent reduction in the rate of NADH oxidation and changes in the composition of ETC complexes, specifically reduced complex I activity [47]. Therefore, it is possible that increased protein-bound NAD(P)H fraction after PDT with Photoditazine is a result of the change in the reduced:oxidized (NADH:NAD+) ratio in the mitochondria. The effects of PDT on tumor metabolism and oxygenation are summarized in Table 1.

A general limitation of the NAD(P)H FLIM approach is that it is unable to report on the specific metabolic pathways underlying the changes in fluorescence decay parameters. So, additional studies using biochemical and molecular assays are needed to uncover the mechanisms of the changes in the optical metabolic metrics upon PDT.

## 4. Materials and Methods

### 4.1. Tumor Spheroids

Multicellular tumor spheroids were obtained from CT26 (murine colorectal cancer) cells stably expressing the phototoxic protein KillerRed fused to histone H2B (KillerRed-H2B). Cancer cells were routinely cultured in Dulbecco’s Modified Eagle Medium DMEM supplemented with 10% fetal bovine serum (FBS), 2 mM L-glutamine, 10 μ/mL penicillin and 10 mg/mL streptomycin. The cells were incubated at 37 °C, 5% CO_2_, and 80% relative humidity.

To generate spheroids, the cells were seeded in 96-well ultra-low attachment round bottom plates in the amount of 100 cells in 200 μL medium. The formation of spheroids with a size of ~300 μm was confirmed in 7 days using light microscopy.

For PDT and subsequent FLIM-microscopy, the 7-day-spheroids were gently transferred onto glass-bottom dishes (8–10 spheroids per dish) in DMEM life medium without phenol red. PDT was performed with a laser MGL-III-593 (CNI, China) at a wavelength of 593 nm. The intensity was 50 mW/cm^2^, exposure time was 25 min, and the light dose was 75 J/cm^2^. Non-irradiated spheroids served as control. The experiment was repeated two times showing reproducible results.

### 4.2. Animal Tumor Model

All the protocols related to experiments on animals were approved by the institutional review board of the Privolzhsky Research Medical University.

**Figure 6:**
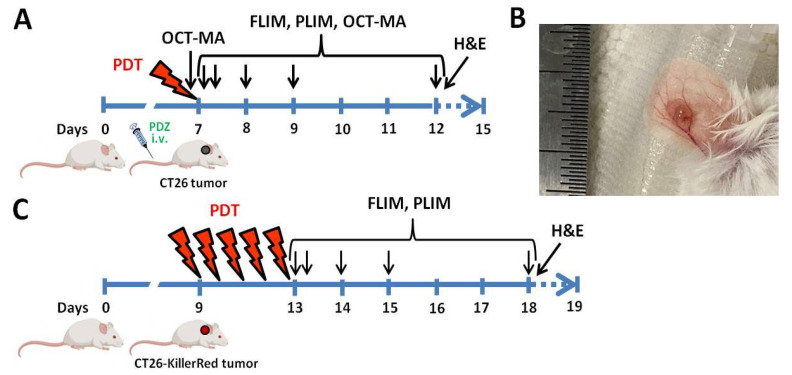
Design of the in vivo study. Schematic overview of the experiments on PDT with Photoditazine (**A**) and KillerRed (**C**). Day 0 is a day of inoculation of CT26 or CT26-KillerRed tumor cells. Photoditazine (PDZ) was injected intravenously (i.v.) in mice with CT26 tumors on Day 7. Laser irradiations of tumors are indicated by red “lightning” signs. Investigations using OCT-MA, NAD(P)H FLIM-microscopy, macro-PLIM and histopathology with H&E are shown by arrows. (**B**) Photograph of the ear tumor model before PDT.

The study was carried out on Balb/c mice, female, weighing 20–22 g, with CT26 or CT26-KillerRed tumors intradermally grafted into the ear (Figure 6).

The CT26 cells and CT26 cells stably expressing KillerRed-H2B were cultured according to standard protocol in a CO_2_ incubator (37° C, 5%, CO_2_ a humid atmosphere) in DMEM (Life Technologies, Carlsbad, CA, USA) supplemented with glutamine, penicillin and streptomycin, and 10% FBS (HyClone, Logan, UT, USA). For inoculation in mice, cells were suspended in phosphate-buffered saline (PBS) at a concentration of 1 × 10^6^ cells/mL and injected intradermally in the ear in the amount of 20 × 10^3^ cells in 20 μL of PBS.

Tumor sizes were measured in two dimensions with a caliper every 2–3 days, starting from day 7 after tumor cell inoculation, and the volume was calculated using the formula V = a × b/2, where a is the length, b is the width of the tumor.

### 4.3. PDT of Mice

Before PDT, animals were anesthetized by intramuscular injection of a mixture of Zoletil (40 mg/kg, 50 μL, Virbac SA, France) and Rometar (10 mg/kg, 10 μL, Spofa, Czech Republic).

Two photosensitizers were used for PDT—Photoditazine (Veta-Grand, Moscow, Russia) and KillerRed.

Photoditazine is N-methyl glucosamine chlorin e6 salt. It is a clinically approved drug used for PDT of malignant tumors of different origin [48]. In the absorption spectra, there are a large absorption band around 400 nm and another band in the red region around 650 nm. Maximum of fluorescence emission is at the wavelength of 662 nm. Photoditazine is supposed to act predominantly via Type II photoreactions, that is generates singlet oxygen (quantum yield of ~0.56) [49]. After intravenous injection, Photoditazine in a tumor targets blood vessels walls and intracellular membrane structures such as the endoplasmic reticulum and Golgi apparatus [50].

PDT with Photoditazine was implemented on day 7 of tumor growth, when the tumor size was ~3–4 mm^3^ (Figure 6A). Photoditazine was injected into the tail vein at a dose of 5 mg/kg, and PDT was carried out 15 min after the injection. Tumors were irradiated with a continuous diode laser (Atkus, St. Petersburg, Russia) operating at the wavelength of 659 nm. The intensity, exposure time and light dose were 120 mW/cm^2^, 12 min, and 86 J/cm^2^, respectively. The predominant vascular response to PDT with Photoditazine within 1 h drug-light interval was demonstrated earlier [34]. Laser power was controlled before each irradiation using a PM100A power meter (Thorlabs, Bergkirchen, Germany).

KillerRed is a dimeric fluorescent protein (excitation maximum 585 nm, emission maximum 610 nm) of the green fluorescent protein family with notable phototoxicity. Upon irradiation with yellow light it generates ROS (presumably superoxide and hydrogen peroxide) in a Type I photodynamic reaction [38]. The key structural features responsible for its unique phototoxic properties are the water-filled channel reaching the chromophore area from the end cap of the β-barrel and the presence of Glu^68^ and Ser^119^ residues, adjacent to the chromophore [51]. Being expressed by the tumor cells that had been transfected with the gene encoded this protein, KillerRed represents a genetically encoded photosensitizer with an exceptional selectivity. Fully genetically encoded nature of KillerRed makes it completely different from chemical photosensitizers in terms of mechanisms of the drug delivery and localization. Among different intracellular targets of KillerRed, fusion with histone H2B showed most pronounced cytotoxic effects in vitro as it interfered with cell division [38].

PDT of KillerRed-expressing tumors started on day 9 of tumor growth, when the tumors reached a size of 3–4 mm^3^. Note, that tumors expressing KillerRed grew slightly slower than tumors generated from their parental cell line CT26. Tumors were exposed to a continuous laser MGL-III-593 (CNI, Qingdao, China) irradiation at a wavelength of 593 nm. The intensity of the laser light was 170 mW/cm^2^. Exposure time was 30 min, and the light dose was 306 J/cm^2^. PDT was performed once a day for 5 days. Design of the experiment is presented on Figure 6. When selecting the treatment mode, we relied on our previous experience on PDT with KillerRed [39].

Tumors that contained a photosensitizer, Photoditazine or KillerRed, but had not been irradiated served as controls.

### 4.4. In Vivo Fluorescence Imaging

To ensure the accumulation/expression of photosensitizers in the tumors and their photobleaching after PDT, fluorescence was recorded in vivo using an IVIS-Spectrum system (Caliper Life Sciences, Hopkinton, MA, USA). Fluorescence of KillerRed was excited at 570 nm (bandwidth 30 nm) and detected at 620 nm (bandwidth 20 nm). Fluorescence of Photoditazine was excited at 640 nm (bandwidth 30 nm) and detected at 720 nm (bandwidth 20 nm). During in vivo imaging, the mice were anesthetized with 2.5% isoflurane. Images were acquired before and immediately after the PDT and analyzed using Living Image 3.2 software (Caliper Life Sciences, Hopkinton, MA, USA). Tumors were selected as regions of interest (ROI) to calculate the average radiant efficiency ((p/s/cm^2^/sr)/(μW/cm^2^)).

### 4.5. FLIM of NAD(P)H

FLIM was performed on two-photon laser scanning microscope LSM 880 (Carl Zeiss, Jena, Germany) equipped with the time-correlated single photon counting (TCSPC) module for time resolution (hybrid detector HPM-100-40; single-photon counting card SPC-150, Becker & Hickl GmbH, Berlin, Germany). Two-photon fluorescence of NAD(P)H was excited at a wavelength of 750 nm with a Ti:Sa femtosecond laser MaiTai HP (Spectra-Physics Inc., Milpitas, CA, USA) and detected in a range of 450–490 nm. Images were acquired using a C-Apochromat 40×/1.3 oil immersion objective. The laser power was ~6 mW. Image collection time was 60 s. To obtain a reasonable accuracy in terms of the fluorescence lifetime evaluation, the number of the photons per decay curve was adjusted to be not less than 5000 using the binning option when necessary.

In spheroids, fluorescence of NAD(P)H was recorded at 6 h and 24 h post-PDT. During image acquisition the spheroids were maintained in the stage top incubator at 37 °C, 5% CO_2_.

The metabolic status of tumor cells in vivo was assessed in 3, 6, 24, 48 h and 5 days after PDT by the fluorescence lifetime of the metabolic cofactor NAD(P)H. 4–6 images were obtained from each tumor at each time point. To acquire images, mice were anesthetized with an injection of Zoletil (40 mg/kg, 50 μL, Virbac SA, Carros, France) and Rometar (10 mg/kg, 10 μL, Spofa, Prague, Czech Republic), placed on a glass coverslip with an ear fixed by a medical tape and mounted in a microscope stage.

The NAD(P)H fluorescence decays were fitted with a bi-exponential function, from which the short and long lifetimes (τ_1_, τ_2_) and their relative contributions (a_1_ and a_2_, respectively, where a_1_ + a_2_ = 100%) were estimated in the SPCImage 8.2 software (Becker & Hickl GmbH, Berlin, Germany). The goodness of the fit, the chi-square, was 0.8 to 1.2. NAD(P)H fluorescence was analyzed in the cytoplasm of each individual cell, which was selected as ROI. A total of 20–40 cells from each tumor were analyzed at each time point.

### 4.6. Macroscopic PLIM

PLIM of the whole mouse tumors in vivo was performed using a confocal FLIM/PLIM macroscanner (Becker & Hickl GmbH, Berlin, Germany), which allows for obtaining time-resolved images from a field of view up of to 18 × 18 mm with a spatial resolution of around 15 μm [23]. The phosphorescent molecular probe BTPDM1 based on iridium (III) complex with benzothienylpyridine containing a cationic dimethylamino group was used for oxygen sensing [27]. Phosphorescence of BTPDM1 was excited in a one-photon mode at a wavelength of 488 nm using a BDL-488-SMC picosecond laser (Becker & Hickl, Berlin, Germany) and detected in the range of 608–682 nm. Laser power incident on the sample was 20 µW. The photon collection time was ~90 s. The number of photons per decay curve was at least 5000. The BTPDM1 solution (12 µM) was injected into the tumor locally, 2–3 injections of 30–50 µL, according to the previously developed protocol [52]. Measurements were carried out 30 min after the injections. The images of tumors were taken in 3, 6, 24, 48 h and 5 days after PDT.

The phosphorescence lifetime of BTPDM1 in tumors was assessed using the SPCImage 8.2 software (Becker & Hickl GmbH, Berlin, Germany). The decay curves were fitted with a monoexponential function and the average phosphorescence lifetime across each tumor was determined.

### 4.7. OCT-Angiography

In the experiments on vascular PDT, the state of the microvasculature in tumors was analyzed using the optical coherence tomography (OCT)-based angiography (OCA). The principle of vascular network imaging is based on determining the temporal variability of the amplitude and phase of the OCT signal in a series of OCT images of the same tissue area. The OCA makes it possible to visualize the perfused blood vessels with transverse spatial resolution of ~15 µm and depth resolution of ~10 µm from a depth of up to ~1.5 mm. The studies were carried out on the spectral multimodal OCT system (BioMedTech, Nizhny Novgorod, Russia) with a central wavelength of 1310 nm, radiation power of 20 mW, the size of the resulting OCT image is 2.4 × 2.4 mm and the scanning speed is 20,000 A-scans/s [53]. OCA images were presented in the form of maximum signal intensity projection—en face image of the vascular network from the entire visualization depth. Using OCA, the structure of the vasculature of the CT26 tumor in mice in vivo was visualized before, immediately (0 h), 6, 24, 48 h and 5 days after PDT.

Perfused vessel density (PVD) was calculated in the original software Anaconda 4.3.1 (Institute of Applied Physics, Nizhny Novgorod, Russia), Python 3.6 (Python Software Foundation, Beaverton, OR, USA) as the number of pixels of all vessel skeletons in the analyzed image area, divided by the total number of pixels in this area as described in Ref. [54].

### 4.8. Histopathology

For histological analysis tumors were taken on either 12th (CT26) or 18th (CT26-KillerRed) days of growth (5 days after PDT). 7-µm-thick paraffin sections were stained with hematoxylin and eosin (H&E) and examined with light microscopy on Leica DM1000 system under 40× magnification. Histopathological examination included visual assessment of tumor blood vessels damage, necrotic areas and cellular morphology.

### 4.9. Statistical Analysis

Data analysis was carried out in the STATISTICA 10.0 software (StatSoft GmbH, Hamburg, Germany). Multiple comparisons were made using ANOVA with the Bonferroni correction. At *p* < 0.05 differences were considered statistically significant. Results presented below are the mean ± standard deviation (SD) or standard error of the mean (SEM).

## 5. Conclusions

Although PDT has proven to be a promising treatment option for cancer, there are still considerable differences in treatment outcomes. More information on underlying physiology is needed to develop new strategies for its improvement. Pre-existing hypoxia and associated glycolytic status of tumors as well as irregular vascular supply are the major determinants of resistance to PDT. At the same time, both oxygenation and metabolic states are affected by PDT, which can promote the acquired resistance. On the other hand, these dynamic transient changes can serve to monitor the efficacy of the treatment and report on the mechanisms of action of photosensitizers. With recent achievements in optical bioimaging, non-invasive monitoring of cellular metabolism, oxygen distribution and vascularization became possible in living mice. In this study, we used a combination of FLIM-microscopy, macro-PLIM and OCA to investigate the influence of PDT on these parameters in the mouse tumor model. We observed that different photosensitizers (KillerRed and Photoditazine) used in a cell death and vascular modes, correspondingly, produced markedly different metabolic changes, presumably due to different degree of PDT-induced hypoxia. The results, presented in this work, are of interest for the search for predictive markers of the effectiveness of therapy and for monitoring the early response of the tumor to treatment.

## Figures and Tables

**Table 1:** Summary of the effects of PDT on tumor metabolism and oxygenation.

	PDT, Photoditazine				
	3 h	6 h	24 h	48 h	5 Days
NAD(P)H	Bound ↑	Bound ↑	Bound ↑	Bound ↑	Bound ↑
O_2_	↓↓	↓↓	=	↑	↓↓
	PDT, KillerRed				
	3 h	6 h	24 h	48 h	5 days
NAD(P)H	Free ↑	Free ↑	=	Bound ↑	Bound ↑
O_2_	↓	↓	↓	↓↓	↑

= equal to control, ↓—lower than control, ↓↓—much lower than control, ↑—higher than control.

## Data Availability

The data are available from the corresponding author upon reasonable request.

## Supplementary Materials

The following supporting information can be downloaded at: https://www.mdpi.com/article/10.3390/ijms25031703/s1.
