# Supplementary material for: Effects of Photodynamic Therapy on Tumor Metabolism and Oxygenation Revealed by Fluorescence and Phosphorescence Lifetime Imaging
Source: Int J Mol Sci. 2024 Jan 30;25(3):1703. doi: 10.3390/ijms25031703 (PMC10855179; doi:10.3390/ijms25031703)
Supplement: Supplementary file 1 [file ijms-25-01703-s001.zip › ijms-2782637-supplementary.pdf]

Supplementary Material

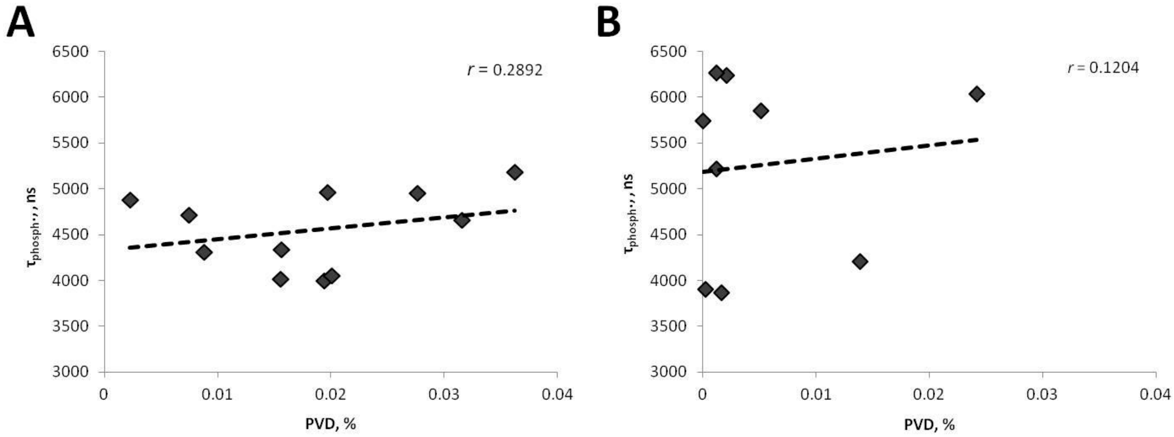

Figure S1. Scatterplot of BTPDM1 phosphorescence lifetimes against perfused vessel densities. Dots are the measurements for individual CT26 tumors in untreated (A) or PDT-treated (B) groups. Dashed lines are the linear approximation. Pearson correlation r is shown on the plots.

Table S1. Fluorescence lifetimes of NAD(P)H in the control tumors and tumors after PDT with either Photoditazine (PDZ) or KillerRed. Mean±SD.

	τ1, ns	τ2, ns	τ1, ns	τ2, ns
	Control		PDT, PDZ	
3 h	0.42±0.01	2.47±0.24	0.41±0.01	2.31±0.21
6 h	0.41±0.01	2.42±0.15	0.42±0.03	2.28±0.11
24 h	0.41±0.02	2.38±0.22	0.43±0.02	2.37±0.14
48 h	0.42±0.03	2.34±0.24	0.41±0.01	2.38±0.22
5 days	0.41±0.01	2.36±0.13	0.43±0.03	2.31±0.31
	Control		PDT, KillerRed	
3 h	0.49±0.01	2.67±0.18	0.51±0.01	2.62±0.31
6 h	0.51±0.02	2.62±0.23	0.48±0.01	2.68±0.21
24 h	0.51±0.03	2.68±0.37	0.53±0.04	2.71±0.22
48 h	0.49±0.02	2.72±0.24	0.49±0.02	2.68±0.22
5 days	0.47±0.03	2.74±0.43	0.45±0.01	2.72±0.35
